# The Association between Adverse Events in the Last 5 Years and the Rate of Breast Cancer

**Published:** 2018-02

**Authors:** Babak RASTEGARIMEHR, Atefeh ZAHEDI, Parvin YAVARI, Mohammad Hassan LOTFI, Moslem TAHERI SOODEJANI

**Affiliations:** 1. Abadan School of Medical Sciences, Abadan, Iran; 2. Dept. of Health and Community Medicine, Shahid Beheshti University of Medical Sciences, Tehran, Iran; 3. Dept. of Biostatistics and Epidemiology, Faculty of Health, Shahid Sadoughi University of Medical Sciences, Yazd, Iran; 4. Dept. of Health, School of Public Health, Kerman University of Medical Sciences, Kerman, Iran

**Keywords:** Breast cancer, Adverse events, Case-control, Iran

## Abstract

**Background:**

This study was conducted in Yazd Province, Central Iran aimed to investigate the relationship between adverse events and breast cancer during 2012–2014.

**Methods:**

Hospital-based case-control study of 150 women with breast cancer and 150 healthy women (did not have breast cancer) was conducted. Sampling was performed in the form of accessibility. Data collection was conducted using questionnaire through interview. The collected data were entered into SPSS for statistical analysis.

**Results:**

The mean age of participants was 51.58 yr. Eight percent of cases and 1.3% of the controls had experienced the sister’s death over the past 5 yr, this difference was statistically significant (*P*=0.03). Factors such as disability due to illness, divorce, unemployment, the second marriage, addiction, ill spouse, child’s problems (such as conflict, unemployment, addiction, legal troubles, illness), taking care of their own parents or their husband’s parents, migration, change of habitat, loss of home, communication problems, job-relevant factors were not significantly different between the two groups (*P*>0.05). Mean of severity of adverse events in both groups was significantly different (8.92±8.29 in the case group, 5.72±5.6 in the control group) (*P*=0.000).

**Conclusion:**

There was no significant relationship between adverse events in the last 5 yr and the risk of breast cancer. Factors such as personality and ability to cope with problems may positively influence this relationship.

## Introduction

Breast cancer is one of the commonest diseases and a major health problem in women worldwide (1). Over 1.5 million women are annually diagnosed with breast cancer, and 502000 people die from this cancer each year (2). The overall five-year survival rate for breast cancer is 78%, ranging from 18% (advanced cancers) to 93% (topical cancers) (3).

In the United States, of all cancers diagnosed among men and women, more than one third are breast cancers (1). Although the overall prevalence of breast cancer is low in Asian countries, the incidence and cause-specific rate in most Asian countries are higher compared to western countries which may be due to increased rate of life expectancy and changed fertility and behavior patterns (4)

In Iran, cancer is the third main cause of death followed by accidents and cardiovascular diseases (5). Breast cancer is the most common cancer among Iranian women (ASR=28.25) (6). This cancer is the most prevalent during the fourth and fifth decades of life in Iranian women, which is one decade earlier compared to other countries (7).

Although some researchers show the strong positive relationship between psychological factors and breast cancer, but another state that, there is not enough evidence for this relationship (8). Psychological factors include negative life events that are experienced by the individuals. Life events and subsequently behavioral and psychological responses positively influence the individual’s daily living and increase the risk of different diseases. Stressful life events increase the risk of mental disorders, acute infections such as cold, and the cause-specific mortality. Moreover, the findings of many studies show the positive relationship between life events and diseases such as cardiovascular diseases, cancer, asthma, and rheumatoid arthritis (9).

Adverse events are physical or psychological events that occur during the daily living and disturb the function of immune system (10). Stress can adversely affect immune system and subsequently which leads to increased risk of tumor growth (11). Emotional factors were one of the main causes of cancer.

Eventually, at the end of nineteenth century, 250 patients admitted to London Hospital were investigated. There was at least one adverse event such as family loss in 156 patients. In spite of lack of control group in this study, it was a comprehensive well-designed study (12). The relationship between adverse events and starting diseases was examined by using a questionnaire.

Since adverse events are positively associated with general health-related disorders (13), and high prevalence of breast cancer in Yazd Province and lack of studies examining the relationship between adverse events and breast cancer in this state, the current study was conducted to examine the relationship between adverse events and breast cancer.

## Methods

Overall, 150 women diagnosed with breast cancer and 150 healthy women (without breast cancer) participated in this hospital-based case-control study. Patients were selected by convenience sampling method of new cases during 2012–2014. The control group was selected from individuals who did not have breast cancer based on the results of mammography or ultrasound. The control and case groups were selected from the same hospital. The inclusion criteria were: 1) Subjects were natives of Yazd or were living in Yazd more than 15 yr. 2) Women whose disease was confirmed by diagnostic tests in case group. 3) No breast cancer in the control group. Exclusion criteria were another cancer apart from breast cancer in case group and people who had a special diet.

After the objectives of the study were defined, patients with informed consent entered the study. Data were collected by face-to-face and telephone interviews. Demographic variables (including age, residence, marital status and occupation), personal habits (such as smoking and passive smoking, duration of exposure to passive smoking, sleep, driving and physical activity), diet variables (including dairy products, oils, fast foods, fried foods, meat, fruits and vegetables in week), reproductive factors (including family history of breast cancer, relative grade of positive family history of breast cancer, history of breast problems, age of menstruation onset, age of menopause, age of first marriage, history of OCP consumption, age of mother at first delivery, number of pregnancy, number of live birth, duration of breastfeeding, history of abortion), adverse events in a 5-yr period, physical activity, body mass index, history of X-ray exposure up to 30 yr, waist to hip ratio. In this paper, only adverse events variables were considered.

Adverse events variables that were associated with psychological consequences of the individuals were extracted from similar studies, questionnaire, views, and experiences of Iranian Psychologists (14–17), then the reliability was approved using Cronbach's alpha (0.76).

The questionnaire was comprised of 39 items (33 questions about adverse events that may be experienced by the individuals over the last 5 yr**,** including family death (father, mother, spouses, children, sibling), divorce, physical or mental illness of spouse, drug addiction and unemployment of spouse, child's physical or mental illnesses, substance abuse, children's unemployment, the presence of debilitating diseases and economic problems. 6 items addressed the history of psychiatric problems, referring to the counseling centers, medication, hospitalization, and suicide. Breast cancer initiates microscopically almost five years before clinical manifestations. Stress at this time may affect the body's immune system and cause growth and spread of cancer cells. Therefore, to determine the role of adverse events in breast cancer progression at least a 5-yr period investigation is required. On the other hand, recall bias may occur if the assessment involves more than this period. Therefore, a period of 5 yr was considered. Cases and controls were matched for age (at the time of interview) in a range of ± 2 yr. Since the new cases were studied, age at the time of interview and age at the time of breast cancer diagnosis are not so different. During the five years prior to the study, different events may have occurred for subjects thus any upsetting event that had affected person, was examined (according to questionnaire). If several events had occurred for the individual, all events were considered.

Data analysis was conducted using SPSS software, descriptive statistics and tests of logistic regression, multiple logistic regression, crude odds ratio and adjusted odds ratio using the techniques of conditional. Those variables that were significant for breast cancer in univariate analysis were examined using multiple logistic regression models and the final model was proposed. If sample size in one box was small, it was merged with the next or previous box in analysis.

## Results

The mean age of participants was 51.58 yr. There were no significant differences between case and control groups due to age adjustment. The median age and standard deviation in case and control groups were 51.56± 10.52 and 51.55± 10.27, respectively.

Distribution of adverse events was assessed. There were no significant differences between variables such as spouse death, death of a child, mother, father, and brother in both groups (*P*>0.05). However, variable, sister's death was significant (*P*=0.01). Twelve (8%) of the patients in case group and two (1.3%) of the subjects in control group experienced sister’s death in the last 5 yr.

There were no significant differences between variables, disability due to illness, divorce, unemployment, remarriage, addiction, sick spouse, child disputes, children's unemployment, child abuse, child legal problems, child's illnesses, parents advocacy, immigration, changing neighborhoods, home lost, communication problems, lack of job satisfaction, job's loss, and job changes (*P*>0.05).

There was a positive relationship between variables, economic problems, conflicts with spouse's family, educational problems in children, legal problems, and marital conflicts and breast cancer in univariate analysis (*P*<0.05) (Table 1).

**Table 1:** Compare the frequency distribution of adverse events factors in case and control groups (univariate analysis)

***Variable***		***Case (%)***	***Control (%)***	***OR***	***P-value***
Death of sister	No	138(92)	148(98.7)	1.81	0.01
	Yes	12(8)	2(1.3)		
Death of brother	No	136(90.7)	144(96)	1.26	0.1
	Yes	14(9.3)	6(4)		
Death of father	No	133(88.7)	125(83.3)	.82	0.057
	Yes	17(11.3)	25(16.7)		
Marital conflicts	No	113(75.3)	130(86.7)	1.43	0.002
	Yes	37(24.7)	20(13.3)		
Legal problems	No	130(86.7)	142(94.7)	2.73	0.02
	Yes	20(13.3)	8(5.3)		
Educational problems of children	No	133(88.7)	143(95.3)	2.61	0.03
	Yes	17(11.3)	7(4.7)		
Conflicts with spouse's family	No	134(89.3)	146(97.3)	4.35	0.01
	Yes	16(10.7)	4(2.7)		
Economic problems	No	89(59.3)	120(80)	2.74	0.000
	Yes	61(40.7)	30(20)		

Approximately, two (1.3%) of the case group and five (3.3%) of the control group had already referred to counseling centers and 28 (18.7%) of case group and 21(14%) of control group reported psychiatric medicine use over the last few years. About 147 of the participants in the case group and two of controls had a history of hospitalization due to psychological problems. Suicide or suicide thoughts in the last 5 yr were nine and one in case and control groups, respectively (Tables 2).

**Table 2:** Comparison of the frequency distribution of psychological factors in case and control groups (univariate analysis)

***Variable***		***Case (%)***	***Control (%)***	***OR***	***P-value***
A history of psychiatric problems	No	120(80)	127(84.7)	1.38	0.28
	Yes	30(20)	23(15.4)		
A history of reference to counseling centers	No	148(98.7)	145(96.7)	2.55	0.25
	Yes	2(1.3)	5(3.3)		
A history of psychiatric medicine consumption	No	122(81.3)	129(86)	0.70	0.27
	Yes	28(18.7)	21(14)		
History of hospitalization	No	3(2)	148(98.7)	0.47	0.33
	Yes	147(98)	2(1.3)		
A history of suicide or suicide thoughts	No	141(94)	149(99.3)	0.33	0.07
	Yes	9(6)	1(0.7)		

The odds ratio for adverse events and history of psychiatric problems variables are shown in Tables 1, 2. Mean of adverse events score was significant between two groups, 8.92 ± 8.29 and 5.72 ± 5.6 in case and control groups, respectively (*P*=0.000).

Finally, all reviewed factors related to adverse events were assessed as one main parameter and the relationship between this parameter and breast cancer was examined. Those who experienced adverse events over the last five years were 1.62 times more likely to develop breast cancer compared to those who never experienced adverse events (Table 3). However, this relationship was not observed after accounting for the effect of other factors such as socioeconomic status, nutrition, and reproductive factors (OR=1.62, p-value=0.25).

**Table 3:** Combination of frequency distribution of adverse events factors in case and control groups (univariate analysis)

***Variable***		***Case (%)***	***Control (%)***	***Odds ratio***	***95%ci***	***P-value***
Adverse events in the last five years	No	66(44)	84(56)	1	-	0.03
	Yes	84(56)	66(44)	1.62	1.02–2.55	

## Discussion

In this study, a significant association between adverse events and breast cancer was observed. Items of adverse events that were significant including death of sister, marital conflicts, legal problems, educational problems of children, conflicts with spouse's family, and economic problems. This association was not significant after consideration of other studied variables. History of psychiatric problems, history of reference to counseling centers, history of psychiatric medicine consumption, history of hospitalization, history of suicide or suicide thoughts were not significant neither in case nor in control groups.

Adverse events can be a starting or progressive factor associated with breast cancer regarding the adverse influence in the function of immune system (11). The effect of life events on breast cancer development depends on the severity of the events and the individual's response to different life events including stress coping strategies and social support (15). The risk of breast cancer was 3.7 times higher in people who experienced stressful events such as family death, spouse death, illness, depression and stress created in daily lives (16). A significant relationship was found between development of breast cancer and physical or mental illness of the children and unemployment of the children. The mean severity of adverse events was similar to our findings (14). Spouse death and financial issues are the most important stressful factors and there was a significant difference between these variables in case and control groups (17). The results of univariate analysis showed a significant relationship between variables, suicide, suicide thoughts, economical problems, conflicts with spouse's family, marital conflicts, and sister’s death. Stressful adverse events increased the risk of breast cancer. However, after accounting for other variables, this relationship was not significant.

The relationship between life events and delayed breast cancer diagnosis was investigated in individuals delaying to see the doctor. No significant relationship between serious adverse life events and delayed breast cancer diagnosis was found. Women who delayed less than 12 wk referring to the doctor reported more non-serious adverse events compared to women who delayed over 12 wk referring to a doctor for diagnosis. Approximately, 72% of the women with less than 12 wk delay in breast cancer diagnosis reported adverse life events one year before the diagnosis (22% serious adverse events and 60% non-serious adverse events) (18).

The mean score of life events was higher in people with breast cancer (12). The majority of people with breast cancer were experienced adverse events such as family death, close friends death or severe illnesses in relatives or friends (19). There was a negative relationship between the wellness and stressful life events in control group, whereas this relationship was not observed in people with breast cancer (20).The risk of breast cancer was increased by 1.05 per events (mean score of life events over the last 5 yr was 4, ranged from 0 to 18) (9).

The weakness of the study related to its design. Restrictions, including bias recall and response bias in case-control studies, are inevitable. To resolve these limitations, all questionnaires were performed by a trained person and with sufficient accuracy. The strength of this study is related to the selection of controls. Thus, ultrasound or mammography was used to make sure that the control group did not have breast cancer.

## Conclusion

There was no significant relationship between 5-yr adverse events and the risk of breast cancer. Factors such as individual's personality and their ability to cope with problems and stressful events may affect this relationship. In other words, adverse events cannot be prevented and normally happen to daily living of individuals, it is very important to learn appropriate coping skills.

## Ethical considerations

Ethical issues (Including plagiarism, informed consent, misconduct, data fabrication and/or falsification, double publication and/or submission, redundancy, etc.) have been completely observed by the authors.

## References

[1] DeSantis C Siegel R Bandi P Jemal A (2011). Breast cancer statistics, 2011. CA Cancer J Clin, 61(6):409–18. 21969133 10.3322/caac.20134

[2] Montazeri A Vahdaninia M Harirchi I (2008). Breast cancer in Iran: need for greater women awareness of warning signs and effective screening methods. Asia Pac Fam Med, 7(1):6. 19099595 10.1186/1447-056X-7-6 PMC2628874

[3] American Cancer Society (2005). Cancer facts and figures 2005. Atlanta (GA): American Cancer Society https://www.cancer.org/content/dam/cancer-org/research/cancer-facts-and-statistics/annual-cancer-facts-and-figures/2005/cancer-facts-and-figures-2005.pdf

[4] Harirchi I Kolahdoozan S Karbakhsh M (2011). Twenty years of breast cancer in Iran: downstaging without a formal screening program. Ann oncol, 22(1):93–7. 20534622 10.1093/annonc/mdq303

[5] Mousavi SM Gouya MM Ramazani R (2009). Cancer incidence and mortality in Iran. Ann oncol, 20(3):556–63. 19073863 10.1093/annonc/mdn642

[6] Department of Cancer, Center of Disease Control, Deputy of Noncommunicable diseases (2009). Report of Iran Cancer Registry. Javan Press Tehran, Iran.

[7] Yavari P (2014). Epidemiology Textbook of Prevalent Diseases in Iran. Volume 3-Cancers. 1st ed GAP Press Tehran, Iran.

[8] Price MA Tennant CC Smith RC (2001). The role of psychosocial factors in the development of breast carcinoma: Part I. The cancer prone personality. Cancer, 91(4):679–85. 11241234

[9] Lillberg K Verkasalo PK Kaprio J (2003). Stressful life events and risk of breast cancer in 10,808 women: a cohort study. Am J Epidemiol, 157(5):415–23. 12615606 10.1093/aje/kwg002

[10] Brown GW Harris T (1978). Social origins of depression. a study of psychiatric disorder in women. Tavistock, London.

[11] Butow PN Hiller JE Price MA (2000). Epidemiological evidence for a relationship between life events, coping style, and personality factors in the development of breast cancer. J Psychosom Res, 49(3):169–81. 11110988 10.1016/s0022-3999(00)00156-2

[12] Ginsberg A Price S Ingram D (1996). Life events and the risk of breast cancer: a case-control study. Eur J Cancer, 32A(12):2049–52. 9014743 10.1016/s0959-8049(96)00275-4

[13] Geyer S (1993). Life events, chronic difficulties and vulnerability factors preceding breast cancer. Soc Sci Med, 37(12):1545–55. 8303339 10.1016/0277-9536(93)90189-b

[14] Ebrahimi M Montazeri A Mehrdad N (2009). Evaluation of events in patients with breast cancer in breast disease center. Breast Disease, 2(1).:30–37. (In Persian)

[15] Tennant C Langeluddecke P Byrne D (1985). The concept of stress. Aust N Z J Psychiatry, 19(2):113–8. 3863602 10.3109/00048678509161308

[16] Kruk J Aboul-Enein HY (2004). Psychological stress and the risk of breast cancer: a case–control study. Cancer Detect Prev, 28(6):399–408. 15582263 10.1016/j.cdp.2004.07.009

[17] Attar Parsaie F Asvadi I (2001). A study of relationship between demographics, life-style, stressful life-events and breast cancer in women. Medical Journal of Tabriz University of Medical Sciences, 35(50):15–21.

[18] Burgess CC Ramirez A Smith P (2000). Do adverse life events and mood disorders influence delayed presentation of breast cancer? J Psychosom Res, 48(2):171–5. 10719134 10.1016/s0022-3999(99)00106-3

[19] Kornblith AB Herndon JE Zuckerman E (2001). Social support as a buffer to the psychological impact of stressful life events in women with breast cancer. Cancer, 91(2):443–54. 11180093 10.1002/1097-0142(20010115)91:2<443::aid-cncr1020>3.0.co;2-z

[20] Ginzburg K Wrensch M Rice T (2008). Breast cancer and psychosocial factors: early stressful life events, social suppor, and well-being. Psychosomatics, 49(5):407–12. 18794509 10.1176/appi.psy.49.5.407

